# Hemodynamic Tolerance of Virtual Reality Intradialysis Exercise Performed during the Last 30 Minutes versus the Beginning of the Hemodialysis Session

**DOI:** 10.3390/healthcare11010079

**Published:** 2022-12-27

**Authors:** Alicia García-Testal, Francisco José Martínez-Olmos, Jose Antonio Gil-Gómez, Víctor López-Tercero, Laura Lahoz-Cano, David Hervás-Marín, Alicia Cana-Poyatos, Rafael García-Maset, Pilar Royo-Maicas, Eva Segura-Ortí

**Affiliations:** 1 Nephrology Service, Hospital de Manises, 46940 Valencia, Spain; 2 Department of Physiotherapy, Universidad Cardenal Herrera-CEU, CEU Universities, 46115 Valencia, Spain; 3 University Institute of Automation and Industrial Technology, Universitat Politècnica de València, 46022 Valencia, Spain; 4 Hemodialysis Unit Nursing, Nephrology Service, Hospital de Manises, 46940 Valencia, Spain; 5 Biostatistics Unit, Instituto de Investigación Sanitaria La Fe, 46026 Valencia, Spain; 6 Research Unit, Nephrology Service, Hospital de Manises, 46001 Valencia, Spain

**Keywords:** exercise, hemodialysis, chronic disease

## Abstract

Background: Exercise improves the physical function of people suffering from chronic kidney disease on hemodialysis (HD). Virtual reality is a new type of intradialysis exercise that has a positive impact on physical function. Intradialysis exercise is recommended during the first 2 h, but its safety in the last part of the dialysis session is unknown. Methods: This was a pilot sub-study of a clinical trial. Several hemodynamic control variables were recorded, including blood pressure, heart rate, and intradialytic hypotensive events. These variables were recorded during three different HD sessions, one HD session at rest, another HD session with exercise during the first two hours, and one HD session with exercise during the last 30 min of dialysis. The intradialysis virtual reality exercise was performed for a maximum of 30 min. Results: During exercise sessions, there was a significant increase in heart rate (6.65 (4.92, 8.39) bpm; *p* < 0.001) and systolic blood pressure (6.25 (0.04,12.47) mmHg; *p* < 0.05). There was no difference in hemodynamic control between the sessions with exercise during the first two hours and the sessions with exercise during the last 30 min. There was no association between intra-dialytic hypotensive events at rest (five events) or exercise at any point (two vs. one event(s), respectively). Conclusion: performing exercise with virtual reality at the end of a hemodialysis session is not associated with hemodynamic instability.

## 1. Introduction

Patients suffering from chronic kidney disease receiving kidney replacement therapy with hemodialysis (HD) present a gradual deterioration in physical function, physical activity levels, and health-related quality of life [1,2]. Exercise can improve fitness, physical function, and some cardiovascular risk factors in this population [3,4]. However, adherence to such exercise programs is low [5,6]. Exercise programs performed during HD treatments achieve higher levels of adherence compared to those performed outside dialysis [7]. It is important to develop strategies to simplify this additional task for HD nursing work and, moreover, to increase patients’ adherence to intradialysis exercise programs. Regarding the timing, exercise implementation is recommended in the first and second hours of the HD session to avoid hemodynamic instability or cramping [8,9,10]. In 2018, Jeong et al., demonstrated the safety of intradialysis exercise performed in the third hour of the HD session [11]. However, its safety at the end of HD treatment is unknown. The safety of intradialysis exercise is a research priority [12]. If the viable time for intradialysis exercise could be extended, and it could be applied at any point during the dialysis session, this would improve its feasibility for several reasons: it could reduce the staff’s work overload and increase hemodialysis patients’ freedom to choose the moment of intradialysis exercise. Additionally, since rehabilitation resources are limited, it would optimize the sharing of resources.

Virtual reality (VR) refers to an interactive computer-generated simulation that presents users with opportunities to engage in environments that appear and feel similar to real-world objects and events. In non-immersive VR, the visualization of virtual elements is achieved through a screen. The interaction is carried out through accessories such as a keyboard, mouse, and microphone or through movement sensors. VR exercise programs are effective in a diverse range of populations, and they have mainly been implemented in neurorehabilitation [13,14,15]. In the field of renal rehabilitation, few studies have explored the effects of VR. It represents a relatively inexpensive and highly applicable tool that can be leveraged to help individuals receiving HD adhere to exercise programs by taking advantage of its leisure-like component and game-like nature. In addition, it is very easy to apply to patients without causing a significant overload for dialysis staff. Thus, it is a feasible intradialysis exercise modality. A few previous studies on VR have shown its benefits for this population through interventions outside of HD [16,17]. Cho and Sohng [16] reported an intervention using Nintendo Wii Fit Plus software three times a week for 8 weeks while patients were waiting for their dialysis at the on-site gym, which resulted in increased strength and skeletal muscle mass and decreased fatigue. Chou et al [17] showed improved levels of overall fatigue, vigor, and motivation and decreased distress and loss of control of mood after 4 weeks of intervention. In 2017, our research group developed the first reported non-immersive VR program for implementation during HD [18]. This pilot study compared two groups, conventional exercise vs. VR, both intradialysis. The game encouraged participants to move their lower limbs, which was converted into movement on the screen through a movement sensor. In this study, our team demonstrated that VR intradialysis exercise improved physical function and maintained participants’ adherence. More recently, we developed a clinical trial involving 12 weeks of intradialysis VR exercise. Our results showed an improvement in several physical function tests, such as 4-m gait speed, the Short Physical Performance Battery, timed up-and-go, one-legged stance test for balance, sit-to-stand 10 and sit-to-stand 60, and the 6-min walking test (6MWT) [19].

Therefore, our aim was to determine the feasibility of an intervention involving non-immersive VR exercise performed during the last 30 min of the hemodialysis session by studying the effect on hemodynamic control.

## 2. Materials and Methods

### 2.1. Design

This was a pilot sub-study from a randomized controlled trial (RCT) (NCT03456414; 6 March 2018) [19]. The participants were recruited from the hospital’s Hemodialysis Unit, and the intervention was implemented between April and September 2018. The inclusion criteria were: having received maintenance dialysis treatment for three months or more; clinical stability; aged over 18 years; and providing informed consent to participate in the study. The exclusion criteria were: myocardial infarction in the previous six weeks; unstable angina during exercise or at rest; lower-limb amputation above the knee without a prosthesis; cerebral vascular disease, such as stroke or transient ischemia; skeletal muscle alterations; respiratory diseases that worsened with exercise; inability to perform functional tests or the planned intervention; and vascular access in the lower limbs. Patients who passed these criteria were informed about the project, and those who agreed to participate provided informed consent.

### 2.2. Intervention

The intervention with the intradialysis VR exercise program was implemented and supervised by a physiotherapist with the support of the HD unit staff. The intradialysis exercise session started with a 5-min warm-up, followed by the VR exercise session for a maximum of 30 min, depending on each patient’s individual fitness level. During the 12 weeks of the RCT, the session was completed during the first two hours of HD. The present pilot sub-study was carried out at the end of the 12 weeks; at this point, the patients performed the exercise session during the last 30 min of a single HD session. To develop the VR exercise program, we adapted a version of the Treasure Hunt game. Treasure Hunt is a non-immersive VR system designed with a game format. Thus, the patients were participating in a game, which may have made their combined exercise HD sessions more enjoyable. In Treasure Hunt, the player must hunt for objects and avoid obstacles by moving their legs. To collect coins and avoid explosives, participants freely move their legs, mainly by raising a lower limb (hip flexion with the knee extended and the foot in a neutral position) and moving both limbs across the screen (hip abduction and adduction with knee extended and foot in neutral position). The difficulty level was graduated according to the characteristics of the participants, and they could change the leg they used to play the game whenever they felt fatigued. The hardware we used was a standard personal computer and monitor screen and Ms Kinect^®^, a movement-detection camera. At the beginning of the session, Treasure Hunt allows the therapist to define the VR intervention by selecting the number of exercise sub-sessions and their duration, as well as the sub-session rest periods. The game difficulty was adaptive, and so the system automatically increased or decreased the level of difficulty depending on each person’s results. An increased difficulty meant that there were more objects to catch and avoid and they appeared and disappeared faster. Once the program was finished, a 5-min cool-down including stretching was performed. The intensity was adapted so that participants rated the perceived exertion between 12 and 15 (6 to 20 scale). If the session was too easy (6–11 on the scale) or too hard (16–20), the physiotherapist re-adjusted the number of sub-sessions and/or their duration. Adherence was defined as the percentage of sessions the participant performed from the total number of sessions offered.

### 2.3. Measurements

The hemodynamic control outcome variables were recorded during three different HD sessions: the first HD session at rest, the second with exercise during the first two hours, and the third with exercise during the last 30 min of dialysis. Systolic and diastolic blood pressure in mmHg and heart rate in beats per minute (bpm) were recorded every 30 min during the HD session, and means and standard deviation (SD) were calculated. These variables were also recorded at the beginning and end of the intradialysis exercise. Intradialytic hypotensive events, defined as hypotensive episodes requiring either saline infusion, the lowering of the ultrafiltration rate (UF), or a reduction in blood flow, were also recorded [20].

### 2.4. Statistical Analysis

Data are presented as mean, standard deviation, median, and first and third quartiles for continuous variables and as relative and absolute frequencies for categorical variables. We analyzed the differences between the participants’ blood pressure and heart rates before and after completing the exercise session using lasso regression [21]. Probability p-values lower than 0.05 were considered statistically significant, and all the analyses were carried out using R software (version 3.2.1) *GNU General Public License*.

### 2.5. Ethics Committee

The Research Ethics Committee approved this research. Informed consent was obtained from all the participants. The present experimental protocol respected the fundamental principles set out in the Declaration of Helsinki, from the Council of Europe Convention on Human Rights and Biomedicine, and in the UNESCO’s Universal Declaration on the Human Genome and Human Rights. It also met the requirements set out in legislation in accordance with the Data Protection Act for biomedical research, personal data protection, and bioethics.

## 3. Results

Forty-five patients were active in the RCT and eligible to participate in the pilot study, of whom thirty-six completed the intervention. Nine patients dropped out of the study due to medical complications (four), receiving a transplant (two), changing HD center (one), and declining to participate (two). The mean age was 68 years (median 73). The descriptive variables are listed in Table 1.

The etiology of renal failure included diabetic nephropathy (27%), glomerulonephritis (16%), vascular kidney disease (including hypertension) (13%), polycystic kidney disease (7%), pyelonephritis (4%), renal hypoplasia (2%), amyloidosis (2%), systemic lupus erythematosus (2%), and unknown (27%).

The total ultrafiltration mean (SD) was 2.02 (0.77) liters per session, with an ultrafiltration rate of 522 (201) ml per hour. There were no significant differences in ultrafiltration in the rest sessions (1.98, 0.77 L), the sessions with exercise in the first two hours (2.06, 0.79 L), and the sessions with exercise in the last 30 min (2.02, 0.79 L).

Exercise intensity was measured through the rate of perceived exertion. The mean perceived exertion achieved during exercise performed within the first two hours was 12.6, and during the exercise performed within the last 30 min of the HD session it was 12.8.

The mean (SD) blood pressure and heart rate determined during one HD session at rest, one HD session with exercise during the first two hours, and one HD session with exercise during the last 30 min are shown in Table 2. The results of the lasso regression analysis showed a negative association between diastolic blood pressure and heart rate for the HD session at rest and the HD session with exercise within the first two hours compared to the HD session with exercise within the last 30 min. The analysis showed no association between systolic blood pressure and rest, exercise at the beginning of the HD session, or exercise at the end of the HD session.

During exercise, both within the first two hours and at the end of the HD session, there was a significant increase in heart rate (bpm) 6.65 (4.92, 8.39) *p* < 0.001 and systolic blood pressure (mmHg) 6.25 (0.04, 12.47) *p* < 0.05. There was no difference in hemodynamic control between the session involving exercise during the first two hours and the session involving exercise during the last 30 min (Table 3). Intradialytic hypotensive events occurred in five patients during HD at rest, two patients during HD with exercise within the first two hours, and one patient during HD with exercise within the last 30 min, but there was no association between any of the groups.

## 4. Discussion

In the present pilot study, we showed that participating in VR intradialysis exercise at the end of HD sessions was safe, and that hemodynamic stability was maintained. This is clinically relevant because it broadens the time window in which HD unit staff can encourage patients to exercise.

### 4.1. Hemodynamic Safety of Intradialysis Exercise

The general recommendation is that intradialysis exercise should be performed in the first two hours of dialysis, because exercise may exacerbate the hemodynamic instability that is most prevalent in the later stages of a dialysis session [9]. Nevertheless, there are not much data to support this recommendation [10]. Exercise causes a decrease in blood volume due to the fluid shift from intra- to extravascular. The mechanisms underlying this are an increase in hydrostatic pressure in the muscle microcirculation and an increase in muscle osmolarity due to the presence of lactate, phosphate, and potassium. The level of hydration is essential to compensate for this effect. There are doubts about whether intradialysis exercise results in hemodynamic instability. Nevertheless, it is known that later on, this mechanism is compensated for by the reflux of proteins into the intravascular space, among other things. In addition, it was proven that in patients who performed intradialysis exercise, this decrease in blood volume was compensated for in a few minutes and did not result in hypotension. The observed effects were an increased heart rate, blood pressure, and cardiac output [22,23,24]. These physiological mechanisms are compatible with the results shown in our study. As Wilund et al. [10] explain, the results of Moore et al. [8] are not enough to justify the contraindication of exercise at the end of the hemodialysis session. Some of their patients had high ultrafiltration rates, and this may have been associated with arterial hypotension. Likewise, it is known that exercise is accompanied by increased blood pressure and followed by hypotension. Dungey et al. [25] assessed these phenomena in patients performing intradialysis exercise in the second hour of the session. They proved that these hemodynamic changes were not accompanied by myocardial damage. The present study verified that hemodynamic stability was maintained even when exercising at the end of the HD session. Other authors have provided results for the safety of intradialysis exercise from the first hour of an HD session until the third hour [11,25,26,27]. Moreover, our results showed a decrease in episodes of arterial hypotension during intradialysis exercise sessions, which were even less frequent if the exercise occurred at the end of the session. Although this decrease did not achieve statistical significance, other authors, such as Rhee et al. [26], found similar results. Future studies should clarify whether intradialysis exercise could be another tool for avoiding intradialysis hypotension [10,27]. The present pilot study showed that VR exercise can be performed at any time during the session without causing instability or clinical complications.

### 4.2. Advantages of Extending the Intradialysis Exercise Time Window

The prevalence of advanced chronic kidney disease and the need for kidney replacement therapy is increasing. However, these patients also experience higher rates of morbidity and mortality compared to the general population. Moreover, the requirement for HD is also accompanied by decreased physical function and health-related quality of life. Considering the nature of this disease and its fatal consequences, the search for solutions to improve the prognosis of these patients must be intensified. Exercise can help to improve these patients’ physical function, independence, and health-related quality of life [2,3,12]. At an HD unit, the nursing staff have multiple tasks to complete. The implementation of intradialysis exercise could overload nursing staff if it is complex, puts patients at risk of instability, or presents an added stressor due to being limited to the first hours of the session. In this study, we used a VR exercise to facilitate the implementation of exercise programs among HD patients and showed that extending the application time window was safe. This may improve the clinical applicability of intradialysis exercise programs.

### 4.3. Non-Immersive Virtual Reality Intradialysis Exercise

Non-immersive VR is a novel modality of intradialysis exercise, which takes advantage of the general technological evolution of society [28]. As stated before, in 2017, our research group implemented the first reported non-immersive VR program during HD [18]. Non-immersive refers to participants interacting with a computer so that their limb movements translate into movements on a screen without the use of VR glasses. In this study, a group of 18 highly motivated patients performed conventional intradialysis exercises for 16 weeks. Then, they were randomly assigned to either continue the conventional exercises or start VR exercises for an additional 4 weeks. The conventional exercise program included both aerobic exercise (cycling, for up to 30 min) and the strength training of the main muscle groups of the lower limbs. The exercises in both the conventional and VR programs were prescribed as “somewhat hard” according to the Borg scale of perceived exertion. The VR group played a game for up to 30 min. Both the conventional and VR groups improved their physical function after 4 weeks (sit-to-stand 10 test, gait speed, and 6-min walk test) with no significant differences between the exercise programs. The adherence during the last 4 weeks of the study was high (conventional 70%, VR 80%). In the present study, a clinical trial involving an intradialytic non-immersive VR exercise program implemented over 12 week, an average adherence rate of more than 70% was achieved, and the intervention was shown to improve patient physical function [19]. Our group has implemented intradialytic programs (combined resistance and aerobic or isolated resistance training) and home-based programs for the last 15 years. The fact that the exercise was perceived as a fun game rather than a challenging exercise session could explain this high adherence to the VR exercise, since these two barriers to exercise have previously been reported for this low-conditioned cohort [5].

In the present study, we verified the technical viability of implementing a VR-guided exercise program within an HD unit and the adaptation of patients to this method, despite their advanced age or sensory limitations in some cases. Because this system does not require a very large financial investment in equipment or personnel, it could be made available in most HD units. Moreover, the generalized implementation of exercise programs among patients on maintenance HD could lead to changes in their prognosis and health-related quality of life, and a global decrease in the societal-level disease burden. VR can facilitate this process.

### 4.4. The Intensity of the Exercise Program

The intensity of the exercise performed by HD patients should not be measured based only on the heart rate achieved during the exercise, because of the autonomic impairment in heart rate control and the common prescription of antichronotropic medications such as beta blockers [2]. Therefore, the intensity of the exercise program was assessed using the rate of perceived exertion. We aimed to achieve a score of at least 12 on the rate of perceived exertion scale (from 6 to 20). The mean intensity was above 12, but we did not record very high rates. Most of the subjects at the hemodialysis units were old and had not exercised much during their lives. It is unrealistic to expect a high intensity of exercise in such a cohort, at least at the start of the exercise program, although some patients rated certain VR exercise sessions above 15 on the scale. The rate of perceived exertion (RPE) is a useful tool for controlling exercise intensity. It is reliable and easy for patients to understand. The RPE has been shown to have good correlation with other physiological measures of work. Training values of 50–85% VO2max correspond to ratings of 12–16 on the Borg RPE scale. The RPE scale (6 to 20) could be used to describe heart rates ranging from 60 to 200 beats/minute [2,29]. However, the RPE also has certain limitations for monitoring exercise intensity [30]. Thus, we present both the RPE and the heart rate control results. The patients in the present study presented an average increase of about 10% in their heart rate during exercise (Table 3). Additionally, this program had a significant impact on physical function, physical activity level, and health-related quality of life, so the intensity was high enough to improve these variables [19,31].

### 4.5. Study Limitations

The limitations of the present study were mainly related to the small sample size and the non-randomization of the groups in terms of exercise time window. Larger, randomized studies are necessary to confirm the present results. Our team is developing a new clinical trial with VR to solve this evidence gap. Moreover, it is possible that the fitness level achieved after 12 weeks of training may favor hemodynamic stability during exercise at the end of the dialysis session. Therefore, it is important to consider that problems may occur in an untrained state. Additionally, these results are exclusively associated with the VR intradialysis exercise.

## 5. Conclusions

In conclusion, performing VR intradialysis exercise during the last 30 min of hemodialysis is not associated with hemodynamic instability. Thus, the feasible period of exercise during a hemodialysis session is longer than previously recommended.

## Figures and Tables

**Table 1:** Patient characteristics at baseline.

	Mean (SD) or Frequency (%)
Age in years	68 (14.64)
Female	18 (40%)
BMI (kg/m^2^)	27.2 (6.97)
Diabetes mellitus	25 (55%)
Smoking habit	11 (24%)
Arteriovenous fistula	39 (86%)
High-flow hemodialysis	27 (60%)
Online hemodiafiltration	16 (35%)
Session duration (minutes)	233.11 (11.74)
Blood flow (mL/min)	378.18 (33.5)
Dialysate flow (mL/min)	582.98 (89.33)
Kt/V sp	1.8 (0.23)
Hemoglobin (g/dL)	11.46 (1.46)
Albumin (g/dL)	3.82 (0.3)

Data shown as the mean and standard deviation (SD) or frequency (%). BMI: body mass index.

**Table 2:** Hemodynamic control during the hemodialysis sessions.

	Rest	Exercise within the First Two Hours	Exercise within the Last 30 Min
Systolic blood pressure (mmHg)	138 (20)	141 (19)	140 (17)
Diastolic blood pressure (mmHg)	60 (11)	61 (11)	62 (11)
Heart rate (beats per minute)	66 (10)	67 (10)	69 (10)

The data are shown as mean and standard deviation (SD) for blood pressure and heart rate determined during full HD session at rest, HD session with exercise within the first two hours, and HD session with exercise within the last 30 min.

**Table 3:** Hemodynamic control during exercise.

	SBP at the Start of Exercise	SBP at the End of Exercise	DBP at the Start of Exercise	DBP at the End of Exercise	HR at the Start of Exercise	HR at the End of Exercise
Exercise within the first two hours	142 (21)	145 (24)	62 (13)	63 (13)	65 (8)	72 (10)
Exercise within the last 30 min	138 (25)	145 (25)	63 (11)	65 (12)	68 (10)	75 (13)

SBP: systolic blood pressure (mmHg), DBP: diastolic blood pressure (mmHg), HR: heart rate (beats per minute). Mean (SD).

## Data Availability

All the data involved in this study are available for consultation upon request through the provided contact address.
